# Drugs and Medicines of North America

**Published:** 1884-06

**Authors:** 


**Drugs and Medicines of North America.** J. U. & C. G. Lloyd, Publishers, 180 Elm Street, Cincinnati, O. Issued quarterly. Price, $1.00 per year.

The first number is before us. The authors announce that it is their intention to take up the entire list of American medicinal plants, issuing their work as a quarterly, and only to subscribers. This number contains three full-page engravings of the plants considered, viz.: Clematis virginiana, Anemone thalictroides and American Pulsatilla, and a full-page microscopic engraving of a section of Clematis virginiana. In addition, there are numerous illustrations in the text. The botanical, chemical and medical histories and descriptions are thoroughly and carefully drawn, all the works that have been written on American drugs being consulted in the preparation of each subject. The medical properties and history are especially interesting to physicians, and in this one work we are presented with all that is known upon each subject. The paper on American Pulsatilla is exceptionally valuable at this period, for preparations of the European plant are now largely imported, and the American species will no doubt supplant the foreign in time to come. The authors state that the following plants will be considered in the immediately succeeding numbers, each article illustrated with a full-page engraving, together with numerous pictures showing the microscopic structure, botanical characteristics, parts used in medicine, sophistications, etc.: Anemone hepatica (liverwort); hydrastis canadensis (golden seal); coptis trifolia (golden thread); actæa alba (white cohosh); cimicifuga racemosa (black snakeroot); xanthorhiza tinctoria (shrubbery yellow root).

